# Early outcomes of on-pump versus off-pump coronary artery bypass grafting

**DOI:** 10.12669/pjms.324.9680

**Published:** 2016

**Authors:** Ghulam Hussain, Hammad Azam, Mirza Ahmad Raza Baig, Naseem Ahmad

**Affiliations:** 1 Ghulam Hussain. FCPS Cardiac Surgery. Assistant Professor of Cardiac Surgery, Ch. Pervaiz Elahi Institute of Cardiology (CPEIC), Multan, Pakistan; 2 Hammad Azam. FCPS (Surgery). Assistant Professor of Cardiac Surgery, Sheikh Zayed Medical College and Hospital, Rahim Yaar Khan, Pakistan; 3 Mirza Ahmad Raza Baig. BS in Cardiac Perfusion. Clinical Perfusionist, Ch. Pervaiz Elahi Institute of Cardiology (CPEIC), Multan, Pakistan; 4 Naseem Ahmad. FCPS Cardiac Surgery. Assistant Professor of Cardiac Surgery, Ch. Pervaiz Elahi Institute of Cardiology (CPEIC), Multan, Pakistan

**Keywords:** Off-pump CABG, On-Pump CABG, Myocardial Infarction

## Abstract

**Objectives:**

To see the early post-operative outcomes of off-pump versus on-pump coronary artery bypass graft surgery.

**Methods:**

This retrospective analytical study was conducted at Ch. Pervaiz Elahi Institute of Cardiology Multan, Pakistan. Our Primary outcome variables were; necessity of inotropic support, nonfatal myocardial infarction, ICU stay, nonfatal stroke, new renal failure requiring dialysis and death within 30 days after operation. There were two groups of patients; Group-I (On-pump group) and Group-II (Off-pump Group). SPSS V17 was used for data analysis. Independent sample t-test and Mann Whitney U test were used to compare quantitative Variables. Chi-square test and Fisher’s exact test were used to analyze qualitative variables. P-value ≤ 0.05 was considered significant.

**Results:**

Three hundred patients were included in this study. There were no significant difference regarding risk factors except hyper-cholestrolemia which was high in off pump group (p-value 0.05). Angiographic and Echocardiographic characteristics e.g. preoperative ejection fraction, LV function grade and severity of CAD was same between the groups. Mortality risk scores and Priority status for surgery were also same. Regarding post-operative outcomes; Post-op CKMB Levels, need and duration of inotropic support, mechanical ventilation time and ICU stay was significantly less in Off-Pump group (p-value 0.001, <0.0001, 0.006, 0.025 and 0.001 resp.). Peri-operative chest drainage was significantly high in On-pump CABG group (p-value 0.027). Incidence of post-op complications was not statistically different between the groups.

**Conclusions:**

At 30 days follow-up, Incidence of myocardial infarction, necessity and duration of inotropic support, ICU stay period and peri-operative bleeding were significantly less in off-pump group. The incidence of neurologic, pulmonary and renal complications was same between the off-pump and on-pump groups.

## INTRODUCTION

Coronary-artery bypass graft (CABG) surgery reduces mortality in patients with severe coronary artery disease.^1^ During the last 30 years, coronary artery bypass grafting (CABG) was performed primarily with the use of cardiopulmonary bypass system (“on pump”) using cardioplegic arrest. Historically, on-pump CABG has shown improvements in ischemic symptoms and prolonged survival in selected patients.^2^,^3^ Using this approach, peri-operative mortality is about 2%, and 5-7% are additional complications along with mortality like myocardial infarction, stroke, and renal failure.^4^

In the mid-1990s, interest developed in performing Off-Pump CABG without the use of cardiopulmonary bypass, in order to reduce postoperative complications associated with the use of cardiopulmonary bypass,^5^,^6^ such as systemic inflammatory response,^7^ cerebral dysfunction,^8^,^9^ myocardial depression, and hemodynamic instability.^10^

Several previous trials have compared off-pump CABG with on-pump CABG.^4^,^11^,^12^ However, none of the previous trials had sufficient power to accurately assess moderate but clinically important differences in rates of death, myocardial infarction, stroke, and renal failure. Furthermore, the skills of the participating surgeons can also influence the outcome of a specific surgical procedure.^13^,^14^

So we conducted this study to compare the early outcomes of off pump versus on pump coronary artery bypass graft surgery to see whether off pump or on pump surgery is better in our center.

## METHODS

It was a retrospective analytical study conducted at Ch. Pervaiz Elahi Institute of Cardiology Multan, Pakistan. The data of patients operated from January 2012 to December 2014 was retrieved from cardiac surgery database of the hospital. Patients having similar pre-operative characteristics were selected for study to minimize the effect of these on post-operative outcomes. Patients who were scheduled to undergo CABG were eligible to participate in this study if they required isolated CABG with median sternotomy.

### Exclusion Criteria

Patients having any clinically significant valvular disease (i.e., moderate, moderate to-severe, or severe valve disease), requiring immediate surgery, with small target vessels (<1.1 mm in internal diameter) or diffuse coronary artery disease were excluded from the study.

Our primary outcomes were; necessity of inotropic support, nonfatal myocardial infarction, ICU stay, nonfatal stroke, new renal failure requiring dialysis and death within 30 days after operation. All deaths in these 30 days were considered to be the result of cardiovascular causes.

### Surgical technique

All procedures were carried out through median sternotomy. For On-pump CABG surgeries, in all patients standard cardiopulmonary bypass (CPB) was established with an ascending aortic straight tip arterial cannula and a two stage single venous cannula in the right atrium. For on-pump procedures, aortic-cross clamp and cardioplegic arrest was applied in every patient. Cold blood cardioplegia was used to arrest the heart. The body temperature during surgery was maintained to about 28-32°C. For off-pump surgery, various stabilization devices were used to provide a motionless surgical field. Conversion from the assigned procedure to the other procedure was performed when clinically necessary.

The necessity of inotropic support on weaning from cardiopulmonary bypass (CPB), and in Intensive Care Unit (ICU) was noted. Peak post-op CK-MB levels were noted twice after 6 & 24 hours of arrival of patient in ICU. Inotropic support was defined as mild if dobutamine was administrated at a rate <5 µg/kg/min, moderate (dobutamine 5-10 µg/kg/min) and high dose (dobutamine>10 µg/kg/min. Renal complication was defined as a rise in serum creatinine levels two folds of preoperative value or the need for renal replacement therapy like hemo-dialysis. The development of significant pleural effusion and pneumothorax and Adult Respiratory Distress Syndrome (ARDS) and Pulmonary Embolism was recorded as pulmonary complication.

### Data Analysis

SPSS Version 17 was used for data analysis. Independent sample t-test and Mann Whitney U test was used to compare quantitative Variables. Chi-square test and Fisher’s exact test (if appropriate) was used to analyzed qualitative variables. P-value ≤ 0.05 was considered significant.

## RESULTS

There was no significant difference regarding demographic characteristics of patients except BMI that was significantly high in patients of group I. Risk factors of Ischemic Heart Disease were same between the two groups. Similarly Angio-graphic, Echocardiographic, mortality risk scores and Priority status for surgery all were same between groups (Table-I). No of grafts applied for revascularization was high in on-pump group, these were 2.92±0.61 versus 2.78±0.68 in off-pump group. But this difference was not statistically significant. Regarding post-operative outcomes, post-op CKMB Levels, need and duration of inotropic support, mechanical ventilation time and ICU stay was significantly less in Off-Pump group. Peri-operative chest drainage was also significantly high in on-pump CABG group (p-value 0.027). Regarding immediate post-operative complications pulmonary complications occurred in 4.7% (7.0) patients in on-pump CABG group and in 2.7% (4.0) patients in Off-pump group. Incidence of neurologic complications was also high in on-pump CABG group (Table-II).

**Table-I:** Base characteristics of Patients under-going On-pump and Off-pump CABG.

Name of Variable		On-pump CABG (Group I)	Off-Pump CABG (Group II)	P-Value
***Demographic Details***				
No. of Patients		150	150	
Age (years)		55.92± 9.26	55.20± 9.96	0.52
Gender (%)	Male	124 (82.7)	116 (77.3)	0.25
	Female	26 (17.3)	34 (22.7)	
Body Mass Index		26.42± 4.76	25.85± 4.20	0.034
Risk Factors of IHD*				
Diabetic history (%)		60 (40.0)	53 (35.3)	0.40
Smoking history (%)		55 (36.7)	63 (42.0)	0.41
History of Hypertension (%)		60 (40.0)	61 (40.7)	0.91
Family History (%)		34 (22.7)	38 (25.3)	0.59
History of hyper-cholestrolemia (%)		15 (10.0)	27 (18.0)	0.05
***Co-morbidities and Peri-operative Data***				
Priority Status	Elective	138 (92.0)	141 (94.0)	0.50
	Urgent	12 (8.0)	9 (6.0)	
Pul. Hypertension		0 (0.00)	3 (2.0)	0.25
Angina Class (CCS)**	Class I	28 (18.7)	31 (20.7)	0.52
	Class II	20 (13.3)	22 (14.7)	
	Class III	95 (63.3)	87 (58.0)	
	Class IV	7 (4.7)	10 (6.7)	
Pre-op Creatinine levels (mg/dl)		0.95± 0.20	0.91± 0.16	0.12
Category of Disease	SVD	9 (6.0)	10 (6.7)	0.89
	DVD	53 (35.3)	56 (37.3)	
	TVD	88 (58.7)	84 (56.0)	
Ejection Fraction (%)		52.43± 9.25	54.00± 8.41	0.13
L.V Function Grades	Grade I	101 (67.3)	110 (73.3)
	Grade II	33 (22.0)	27 (18.0)	
	Grade III	16 (10.7)	13 (8.7)	
Parsonnet score		4.04± 3.31	3.57± 3.06	0.19
Add-euro Score		1.17± 1.16	1.09± 1.09	0.57
Log-euro Score		1.37± 0.58	1.30± 0.56	0.29

* IHD= Ischemic Heart Disease,

** CCS= Canadian Cardiovascular Society.

**Table-II:** Comparison of Operative and Post-operative outcomes.

Name of Variable		On-pump CABG (Group I)	Off-Pump CABG (Group II)	P-Value
No. of Grafts (Mean±S.D)		2.92±0.61	2.78± 0.68	0.06
Chest Drainage		661.95± 381.67	581.84± 219.68	0.027
Post-op CKMB* Levels (IU)		57.69± 38.57	30.27± 21.85	0.001
Duration of Support (hours)		11.41± 15.95	6.74± 12.41	0.006
Ventilation time (hours)		6.81± 6.97	5.34± 3.79	0.025
ICU Stay (hours)		42.67± 23.44	35.02± 12.15	0.001
Hospital stay (days)		7.35± 2.87	7.11± 1.97	0.401
IABP** (%)		2.0 (1.3)	1.0 (0.7)	0.562
Operative Mortality (%)		2.0 (1.3)	0 (0.00)	0.16
Inotropic Support (%)	Mild	89 (59.3)	34 (22.7)	<0.0001
	Moderate	32 (21.3)	6.0 (4.0)	
	High Dose	2.0 (1.3)	0.0 (0.00)	
	Nil	27 (18.0)	110 (73.3)	
Pul. Complications (%)		7.0 (4.7)	4 (2.7)	0.36
Neurologic complications	Brain death	1 (0.7)	0.0 (0.00)	0.62
	Nil	147 (98.0)	149 (99.3)	
	Acute Confusional State	2 (1.3)	1 (0.7)	
Renal Complications (%)		2 (1.3)	2 (1.3)	1.00

* CKMB=Creatinine Kinase Myocardial Band, IABP= Intra-aortic Balloon Pump.

## DISCUSSION

Trend of Off-pump CABG has been increasingly in use in Western countries since the early 1990s, when Benetti^5^ and Buffolo^15^ demonstrated excellent benefits possibly associated with the avoidance of cardiopulmonary bypass.^16^ About 15% to 20% of CABG procedures in the Western world and most CABG operations in Asia are performed off-pump without the use of cardiopulmonary bypass. Multiple randomized trials, observational studies have compared peri-operative mortality, and surgical complications and outcomes, for Off pump CABG and on-pump CABG.^16^ Many studies, typically conducted in low-risk patients, found no significant differences in peri-operative mortality, but did find a reduced need for blood transfusions, ventilation and shorter hospital stay times with off pump CABG.^17^,^18^ Meta-analyses reports also showed similar results,^19^,^20^ Large observational studies reported significant reductions in peri-operative mortality and morbidity, and similar peri-operative outcomes.^21^-^23^ Whereas many other studies have concluded that off pump-CABG is associated with a high risk of long term mortality; probably this may be due to high risk of incomplete revascularization in off pump CABG group. As many studies have shown lower rates of complete revascularization in off pump CABG patients.^24^,^25^ In our study, the number of grafts were less in off-pump group but this difference was statistically not significant (p-value 0.06) so it can be neglected.

Off pump CABG has demonstrated decreased blood loss and need for transfusion, decreased length of stay, less myocardial enzyme leak, less inflammation, and less renal and neurologic impairment in addition to decreased cost.^12^,^26^,^27^ Reston and colleges^28^ in their meta-analysis report of comparing short term and midterm outcomes of off-pump CABG, found that off pump CABG is associated with reduced length of hospital stay, operative morbidity, and operative mortality as compared with on pump CABG.

Our study supports the results of these studies as in our study, peri-operative chest drainage was significantly low in Off-Pump Group. We found significant difference regarding post-op CK-MB levels, need and duration of inotropic support, mechanical ventilation time and ICU stay time, all were high in on-pump CABG group. However we do not find any significant difference in immediate post-operative complications in both groups pulmonary and neurologic complications were a bit high in on-pump CABG group but this difference was not statistically significant. In our study, operative mortality was high in on-pump CABG group, there were two deaths in this group in a one month follow up in this group but there was no mortality in Off-pump CABG group.

The results of our study revealed that off pump CABG is a safe and better option as regards to early extubation, respiratory complications and early recovery and a lower risk of short term mortality.

## CONCLUSIONS

At 30 days follow-up, Incidence of myocardial infarction, necessity and duration of inotropic support, ICU stay period and peri-operative bleeding were significantly less in off-pump group. And the incidence of neurologic, pulmonary and renal complications was same between the off-pump and on-pump groups.
